# Different features for different races: Tracking the eyes of Asian, Black, and White participants viewing Asian, Black, and White Faces

**DOI:** 10.1371/journal.pone.0310638

**Published:** 2024-09-18

**Authors:** E. Darcy Burgund, Yiyang Zhao, Inaya N. Laubach, Eyerusalem F. Abebaw

**Affiliations:** Department of Psychology, Macalester College, Saint Paul, Minnesota, United States of America; Lamar University, UNITED STATES OF AMERICA

## Abstract

The own-race bias (ORB) is an effect in which humans remember faces from their own race better than faces from another race. Where people look when processing faces of different races plays a role in this effect, but the exact relationship between looking and the ORB is debated. One perspective is that the same facial features are important for memory for faces of all races and the ORB emerges when people look longer at the useful features for own- than other-race faces. Another perspective is that different facial features are useful for faces of different races and the ORB emerges when people look longer at features that are useful for their own race than at features that are useful for other-race faces. The present study aimed to discriminate these perspectives by examining looking patterns in Asian, Black, and White participants while they learned and later recognized Asian, Black, and White faces. Regardless of their race, participants looked at different facial features depending on the race of the face. In addition, different features were useful for memory depending on the race of the face. As such, results are in line with the perspective that different facial features are useful for different race faces.

## Introduction

Human faces are categorized into different racial groups based on skin tone and physiognomy. The concept of “race” is distinct from ethnicity (shared culture, e.g., language, dress, food) and nationality (country citizenship) and refers only to the way someone looks. This concept nonetheless has enormous influence on many psychological processes. One such influence of race on processing is the tendency to remember faces of one’s own race to a greater extent than faces of another race [1]. This effect, often referred to as the own-race bias (ORB), has consequences for daily life, leading to less socialization among members of different races and greater isolation within groups for people of minority races [2]. As such, insights to the mechanisms underlying the ORB are important for increased understanding of face processing and memory and of an effect that has important real-world implications.

Studies examining where people look when viewing own and other-race faces have informed theories of the mechanism underlying the ORB. In a seminal study by Goldinger and colleagues [3] in which White (of European descent) and East Asian participants exhibited an own-race bias for White and East Asian faces, participants looked at the eyes of faces of their own race more than the eyes of faces of the other race, and at the nose and mouth of faces of the other race more than the nose and mouth of faces of their own race. This finding has been replicated with White participants viewing White and East Asian faces [4], White and Black (of African descent) faces [5–7], and White, Black, and East Asian faces [8], and suggests that looking at the eyes is useful for all race faces and people look at the eyes for varying amounts of time depending on whether the face is from their own or another race. Reasons for why people might look differently at own compared to other-race faces include attention to race-stereotypical features in other-race faces and differential motivation for own compared to other-race faces (see, e.g., [9]). Regardless of the reason however, this account of the ORB argues that the same information is useful for all race faces, and the ORB emerges when people look at this useful information more for own than other-race faces.

In contrast to this idea, other studies suggest that different facial features are useful depending on the race of the face, with the eyes being more informative for White faces and the nose and mouth being more informative for East Asian and Black faces [10–14]. According to this perspective, people become experts at extracting the information that is useful for the type of faces they process most often (typically faces of their own race), and the ORB emerges when they rely on this information for other-race faces instead of information that would be useful for that race face. For example, in a study in which Black and White participants exhibited the ORB, Black participants looked at the nose and mouth of both Black and White faces more than White participants did, while White participants looked at the eyes of both Black and White faces more than Black participants did [12].

Recent research suggests that the influence of looking on the ORB lies somewhere between the extremes of these different perspectives. Burgund [15] tracked the eyes of East Asian and White participants while they learned and later recognized East Asian, White, and Black faces. In line with the idea that different features are used for different race faces, East Asian and White participants relied on the same strategy of looking at the eyes of East Asian and White faces longer than the eyes of Black faces and at the nose/mouth area of Black faces longer than the nose/mouth area of East Asian and White faces. Also in line with this idea, longer looking at the eyes of East Asian and White faces predicted greater memory for East Asian and White faces, and longer looking at the nose/mouth area of Black faces predicted greater memory for Black faces. Critically however, even though East Asian and White participants did not look at the eyes of Black faces for as long as they looked at the eyes of East Asian and White faces, looking at the eyes of Black faces positively predicted memory for Black faces, indicating that eye looking is important for remembering Black faces just as it is important for remembering East Asian and White faces. As such, results from Burgund [15] provide support for a hybrid model of face memory in which the eyes are important for remembering all race faces and the nose/mouth area is important for remembering Black faces.

It is not clear from the Burgund [15] study, however, whether the pattern of looking exhibited by East Asian and White participants for Black faces was due to different features being differentially useful for Black faces or to the fact that the faces were “other race” for all the participants. Indeed, East Asian and White participants had greater memory for faces of their own race than for Black faces, and looked more at the eyes and less at the nose/mouth of faces of their own race than Black faces, which matches the theory that the eyes are useful for all faces and the ORB emerges when people look at the eyes more for own-race than other-race faces (e.g., [3–8]). However, results from the East Asian and White faces suggest that looking patterns are due the utility of different features for East Asian and White compared to Black faces, rather than to the fact that Black faces were “other race”, because participants looked at East Asian and White faces similarly even though one of the face types was “other race” for each group (East Asian faces for White participants; White faces for East Asian participants). This is in line with the perspective that different features are useful for different race faces (e.g., [10–14,16]).

These possible explanations for the different looking patterns for East Asian and White compared to Black faces cannot be distinguished without examining looking patterns in Black participants for whom Black faces are “own” rather than “other” race. Thus, the present study examined looking patterns of Black participants, as well as East Asian and White participants, while they learned and then recognized Black, East Asian, and White faces. If reduced eye looking and increased nose/mouth looking for Black compared to East Asian and White faces is due to Black faces being “other race” for East Asian and White participants, then Black participants should not exhibit this pattern and should instead exhibit greater eye looking and less nose/mouth looking for Black faces than East Asian and White participants exhibit. In contrast, if reduced eye looking and increased nose/mouth looking for Black compared to East Asian and White faces is due to differential utility of these features for Black compared to East Asian and White faces, then Black participants should rely on a similar strategy as East Asian and White participants with greater eye looking for East Asian and White faces compared to Black faces and greater nose/mouth looking for Black faces compared to East Asian and White faces.

The present study also measured participants’ contact with Black, East Asian, and White people during childhood, teenage years, and currently, as experience with other-race faces is argued to be a crucial contributor to the ORB [17–20], especially during childhood [21,22]. The few eye-tracking studies of the ORB that have measured previous contact have not observed effects of contact on looking behavior [15,23]. Nonetheless, it is useful to measure and control for contact due to the substantial role of previous experience in face processing more generally [24], as well as to increase the number of ORB eye-tracking studies that have included this variable.

## Method

### Ethics statement

The present experiment was approved by the Institutional Review Board at Macalester College (Approval number: 22050). Participants provided written consent in accordance with the guidelines established by the Code of Ethics of the World Medical Association (Declaration of Helsinki).

### Participants

Data collection occurred between May 31, 2022 and May 2, 2023. Eighty-five participants took part in the experimental protocol, however 4 were excluded due to their chance performance on the memory test (48–52%) leaving a total of 81 participants included in the study. All were Macalester College students between 18–24 years old (*M* = 20.35; *SD* = 1.17). Asian participants (*N* = 28; 18 female, 8 male, 1 nonbinary) identified as racially Asian and had lived the majority of their lives (13–21 years; *M* = 17.36; *SD* = 2.20) in an East Asian country (China [82%], Japan [7%], Myanmar, Philippines, and Vietnam [11%]). Black participants (*N* = 25; 21 female, 4 male) identified as racially Black and had lived the majority of their lives (10–21 years; *M* = 18.10; *SD* = 2.98) in the United States (96%) or Canada (4%). White participants (*N* = 28; 17 female, 8 male, 3 nonbinary) identified as racially White and had lived the majority of their lives (19–23 years; *M* = 20.36; *SD* = .99) in the United States. Participants were compensated $15 for taking part in the study.

### Design

The experiment employed a 3 x 3 mixed-factorial design in which participant race (Asian vs. Black vs. White) was a between-subjects independent variable and face race (Asian vs. Black vs. White) was a within-subjects independent variable. For the analysis of looking times, feature (eyes vs. nose vs. mouth) was also included as a within-subjects independent variable for a 3 x 3 x 3 mixed-factorial design. A sensitivity analysis using G*Power 3.1 [25] revealed that, with an alpha of .05 and 80% power, 81 participants is sufficient to detect a medium-large effect size of η_p_^2^ = .108 in the 3 x 3 design and η_p_^2^ = .138 in the 3 x 3 x 3 design. Dependent variables were childhood contact, looking time during the learning and recognition memory phases, and recognition memory performance.

### Materials

Stimuli were 60 emotionally neutral facial images of adults taken from the Multi-Racial Mega-Resolution database (MR2) [26], which includes images of African, East Asian, and European faces. Twenty East Asian faces (12 female, 8 male) were selected to serve as Asian faces in the present study; 20 African faces (10 female, 10 male) served as Black faces; and 20 European faces (10 female, 10 male) served as White faces. The numbers of female and male East Asian faces differed from those for Black and White faces due to limitations of the MR2 database, which only includes 8 male East Asian faces. All images were cropped into an oval shape that excluded the hair and ears, converted to grayscale on a white background, set to equal mean luminance, and scaled to a height of 425 pixels, which, when viewed from the 60 cm distance used in the present study, approximates the size of a real face (around 20 cm in height) viewed from a distance of about 80 cm during a typical human interaction (approximately 14˚ of vertical visual angle).

Contact with Asian, Black, and White people during childhood, teenage years, and currently was measured using questions adapted from McKone et al. [21]. For childhood, participants were instructed: “Think about when you were between 0–10 years old. Estimate what percentage (0–100%) of your ___ were each of the following races: East or Southeast Asian; Black; White”, with ___ referring to “classmates at school”, “friends”, or “neighbors” in three different questions. Participants were then asked these questions for teenage years (“Think about when you were between 10–17 years old…”) and currently (“Think about now…”). Classmates, friends, and neighbors questions had excellent reliability for each race during childhood (Cronbach’s α = .987 for East or Southeast Asian, Cronbach’s α = .941 for Black, and Cronbach’s α = .968 for White) and during teenage years (Cronbach’s α = .973 for East or Southeast Asian, Cronbach’s α = .891 for Black, and Cronbach’s α = .922 for White), but low reliability for current (all Cronbach’s α‘s < .526). As shown in S1 Fig, this low reliability between questions during the current time period comes from a difference between responses to the friends question and the classmates and neighbors questions in Asian and Black participants who reported more friends of their own race compared to White, and more White classmates and neighbors compared to classmates and neighbors of their own race. Responses to classmates, friends, and neighbors questions were averaged for each race for each time period to create “contact” scores for each participant.

### Procedure

Participants began the study by completing a consent form that described the nature of the tasks they would perform. After consenting to take part in the study, participants placed their chins in a chin-rest to keep their eyes approximately 60 cm from the computer screen, and eye tracking began. Eye movements were recorded using a desktop-mounted EyeLink 1000 eye tracker. Eye fixations were calibrated before the learning and recognition memory phases of the experiment using a 9-point fixation procedure, which was repeated as necessary until the calibration criterion was reached. Automatic drift correction was performed before each trial during both the learning and recognition memory phases of the experiment.

During the learning phase of the experiment, 30 faces (10 Asian, 10 Black, 10 White) were presented in a random order, with 6 filler faces (2 per race) presented (3 at the beginning and 3 at the end) in order to attenuate primacy and recency effects. Each learning phase trial consisted of a fixation cross at the center of the screen for 1500 ms followed by a face presented in one of the four quadrants of the screen for 5000 ms. Participants were instructed that they should try to learn and remember each face for a memory test that would happen later.

After the learning phase, participants completed a set of mazes with pencil and paper for 5 minutes as a distractor task. Once the 5 minutes had ended, eye fixations were recalibrated, and the recognition memory phase began. During this phase, the 30 learned faces were presented again, intermixed with a set of 30 new faces (10 per race). Across participants, all faces appeared equally often as learned and new. Each recognition memory trial consisted of a fixation cross at the center of the screen for 1500 ms followed by a face presented in one of the four quadrants of the screen. Participants were instructed to decide whether the face had been seen in the previous learning phase or not and to indicate their response by pushing a key on the keyboard. The face remained on the screen until the participant responded.

After the recognition memory phase, participants completed a questionnaire in which they provided basic demographic information and responded to the childhood contact questions. Participants were then debriefed, thanked, and paid for their time. The entire study took between 30–45 minutes depending on how much time was needed for eye calibration.

## Results

### Contact

**Fig 1:**
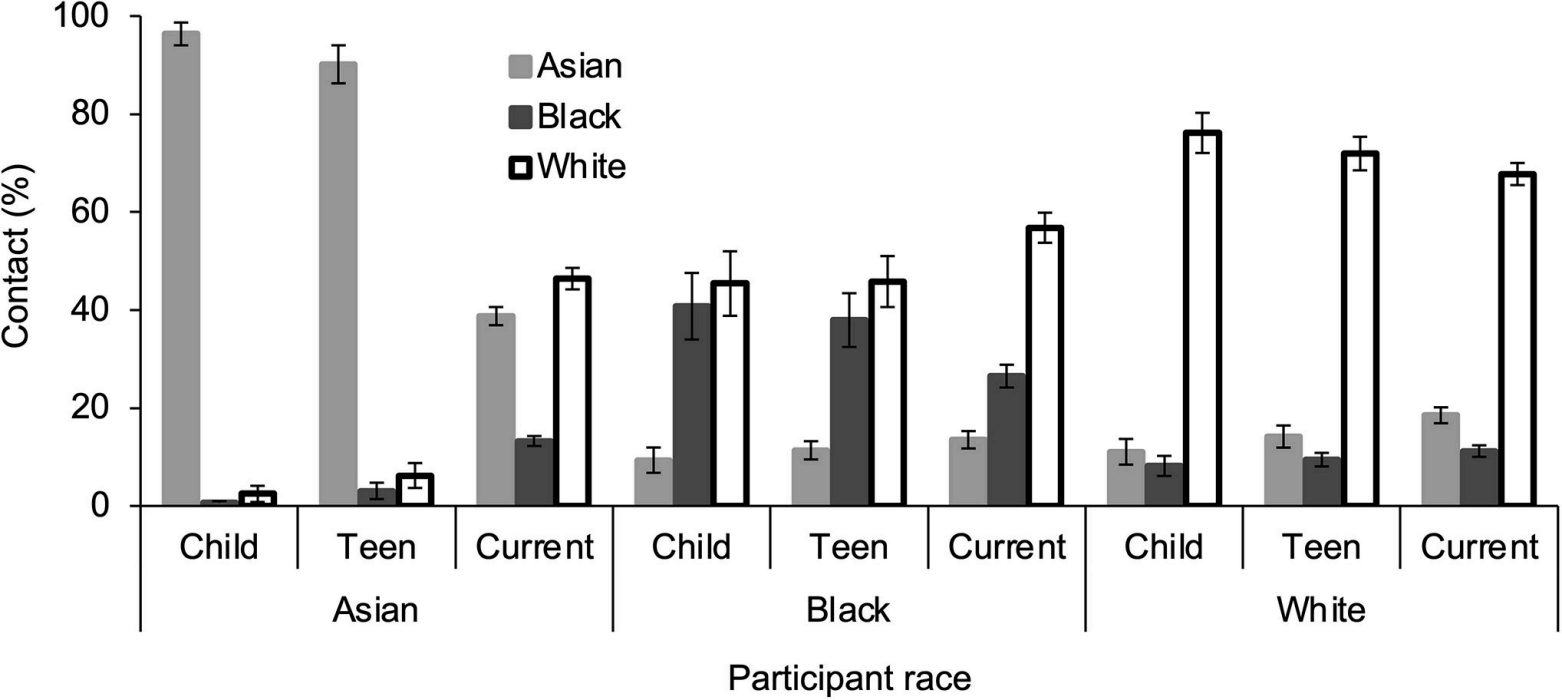
Contact with different races during different time periods. Vertical bars indicate standard error of the mean.

Contact with different races was analyzed in a 3 x 3 x 3 repeated-measures analysis of variance (ANOVA) with target race (Asian vs. Black vs. White) and time period (childhood vs. teenage vs. current) as within-subjects variables and participant race (Asian vs. Black vs. White) as a between-subjects variable. Significant two-way interactions of target race x time period, *F*(4, 312) = 23.88, *p* < .001, η_p_^2^ = .234, 95% CI [.174, .287], and target race x participant race, *F*(4, 312) = 156.22, *p* < .001, η_p_^2^ = .667, 95% CI [.618, .707], as well as the three-way interaction of target race x time period x participant race, *F*(8, 312) = 33.48, *p* < .001, η_p_^2^ = .462, 95% CI [.405, .511], were observed (see Fig 1). The three-way interaction was examined with two-way repeated-measures ANOVAs of target race x time period for each participant race.

The two-way analysis for Asian participants revealed a main effect of target race, *F*(2, 54) = 464.99, *p* < .001, η_p_^2^ = .945, 95% CI [.922, .958], in which Asian participants had greater contact with members of the Asian race (*M* = 75%, *SD* = 10%) than the White race (*M* = 18%, *SD* = 8%), *t*(27) = 17.57, *p* < .001, *d* = 6.30, 95% CI [4.44, 7.56], which was greater than their contact with members of the Black race (*M* = 6%, *SD* = 3%), *t*(27) = 9.64, *p* < .001, *d* = 1.85, 95% CI [1.33, 2.37]. The effect of target race was moderated by an interaction with time period, *F*(4, 108) = 139.21, *p* < .001, η_p_^2^ = .838, 95% CI [.794, .867], in which contact with members of the Asian race was greater than contact with members of Black and White races during childhood (Asian vs. Black: *t*(27) = 36.77, *p* < .001, *d* = 6.76, 95% CI [4.93, 8.59]; Asian vs. White: *t*(27) = 23.71, *p* < .001, *d* = 4.48, 95% CI [3.23, 5.72]) and teenage years (Asian vs. Black: *t*(27) = 16.34, *p* < .001, *d* = 3.09, 95% CI [2.18, 3.98]; Asian vs. White: *t*(27) = 13.26, *p* < .001, *d* = 2.51, 95% CI [1.74, 3.26]), but not during the current time period. During the current period, Asian participants reported greater contact with members of both Asian and White races compared to members of the Black race (Asian vs. Black: *t*(27) = 12.18, *p* < .001, *d* = 2.30, 95% CI [1.58, 3.01]; White vs. Black: *t*(27) = 12.34, *p* < .001, *d* = 2.33, 95% CI [1.60, 3.05]), and no difference between Asian and White races, *t*(27) = 1.90, *p* = .069, *d* = .358, 95% CI [-.027, .738].

The analysis of target race x time period for Black participants revealed a main effect of target race, *F*(2, 48) = 22.00, *p* < .001, η_p_^2^ = .478, 95% CI [.281, .616], in which Black participants had less contact with members of the Asian race (*M* = 11%, *SD* = 10%) than the Black race (*M* = 35%, *SD* = 19%), *t*(24) = 5.12, *p* < .001, *d* = 1.96, 95% CI [1.26, 2.67], and White race (*M* = 49%, *SD* = 20%), *t*(24) = 7.77, *p* < .001, *d* = 3.39, 95% CI [2.74, 4.04], which did not differ, *t*(24) = 1.92, *p* = .066, *d* = .707, 95% CI [.053, 1.36]. The effect of target race was moderated by an interaction with time period, *F*(4, 96) = 2.53, *p* = .046, η_p_^2^ = .095, 95% CI [.000, .180], in which contact with members of the Asian race was less than contact with members of Black and White races during childhood (Asian vs. Black: *t*(24) = 3.98, *p* < .001, *d* = .796, 95% CI [.338, 1.24]; Asian vs. White: *t*(24) = 4.57, *p* < .001, *d* = .915, 95% CI [.439, 1.38]) and teenage years (Asian vs. Black: *t*(24) = 4.15, *p* < .001, *d* = .830, 95% CI [.367, 1.28]; Asian vs. White: *t*(24) = 5.83, *p* < .001, *d* = 1.17, 95% CI [.648, 1.67]), but not during the current time period. During the current period, Black participants reported greater contact with members of the White race compared to the Black race, *t*(24) = 5.78, *p* < .001, *d* = 1.16, 95% CI [.639, 1.66], and greater contact with members of the Black race compared to the Asian race, *t*(24) = 4.66, *p* < .001, *d* = .931, 95% CI [.453, 1.40].

The analysis of target race x time period for White participants revealed a main effect of target race, *F*(2, 54) = 276.84, *p* < .001, η_p_^2^ = .909, 95% CI [.878, .928], in which White participants had greater contact with members of the White race (*M* = 72%, *SD* = 14%) than the Asian race (*M* = 15%, *SD* = 8%), *t*(27) = 15.69, *p* < .001, *d* = 4.04, 95% CI [3.56, 4.52], which was greater than the Black race (*M* = 10%, *SD* = 5%), *t*(27) = 2.62, *p* = .014, *d* = .513, 95% CI [.086, 939]. The effect of target race was moderated by an interaction with time period, *F*(4, 108) = 3.02, *p* = .012, η_p_^2^ = .100, 95% CI [.041, .184], in which contact with members of the White race was greater than contact with members of Asian and Black races during childhood (White vs. Asian: *t*(27) = 11.05, *p* < .001, *d* = 2.09, 95% CI [1.42, 2.75]; White vs. Black: *t*(27) = 13.33, *p* < .001, *d* = 2.52, 95% CI [1.75, 3.28]) and teenage years (White vs. Asian: *t*(27) = 11.61, *p* < .001, *d* = 2.19, 95% CI [1.50, 2.88]; White vs. Black: *t*(27) = 16.49, *p* < .001, *d* = 3.12, 95% CI [2.21, 4.02]), but during the current time period, White participants not only had greater contact with members of the White race compared to Asian race, *t*(27) = 13.48, *p* < .001, *d* = 2.55, 95% CI [1.77, 3.31], but also had greater contact with members of the Asian race than the Black race, *t*(27) = 4.53, *p* < .001, *d* = .856, 95% CI [.416, 1.29].

The 3 x 3 x 3 repeated-measures ANOVA also revealed a main effect of target race, *F*(2, 156) = 90.58, *p* < .001, η_p_^2^ = .539, 95% CI [.477, .594], in which participants had less experience with members of the Black race (*M* = 16%, *SD* = 17%) than the Asian race (*M* = 35%, *SD* = 31%), *t*(80) = 3.94, *p* < .001, *d* = .848, 95% CI [.511, 1.19], and White race (*M* = 46%, *SD* = 27%), *t*(80) = 8.38, *p* < .001, *d* = 1.96, 95% CI [1.53, 2.40], which were marginally different, *t*(80) = 1.96, *p* = .053, *d* = .346, 95% CI [.059, .633]. The main effect of participant race was not significant, *F*(2, 78) = 1.40, *p* = .253, η_p_^2^ = .034, 95% CI [.000, .091].

### Looking time

Prior to analyzing the looking time data, non-overlapping left eye, right eye, nose, and mouth areas of interest were defined for each of the 60 faces used in the study using similar parameters to previous research [3–5,15]. All areas were rectangular; eye areas were the width of each eye and extended vertically from the bottom of the eye to the top of the eye brow; nose areas were the width of the widest part of the nose and extended vertically from the bottom of the nose to the midpoint of the nose bridge; mouth areas were the width and height of the mouth. The amount of time in milliseconds that each participant looked at each area of interest was extracted for each face using the default algorithm provided by the EyeLink 1000 system; times for the left- and right-eye areas were summed to create an “eyes” condition.

**Fig 2:**
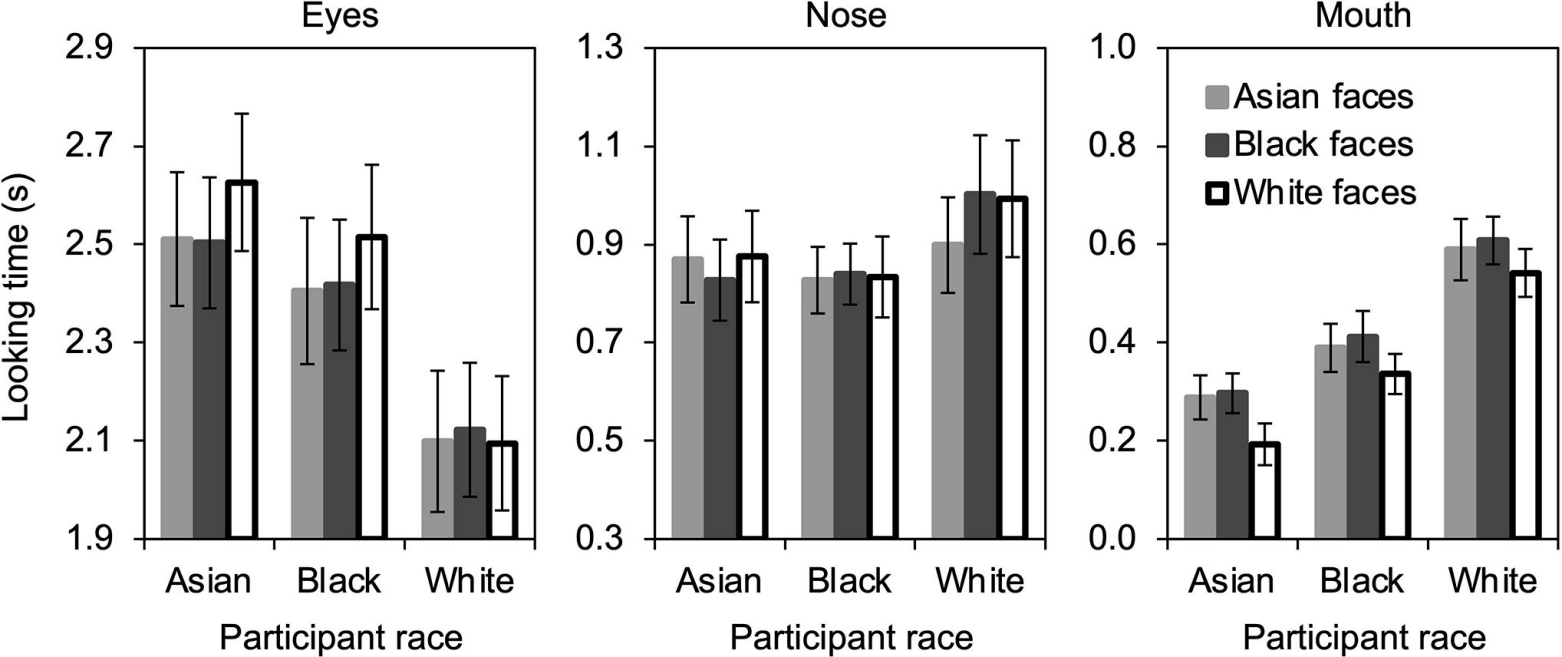
Looking time (s) during the learning phase. Vertical bars indicate standard error of the mean.

Looking times during the learning phase of the experiment were analyzed in a 3 x 3 x 3 repeated-measures ANOVA with participant race (Asian vs. Black vs. White) as a between-subjects variable, and face race (Asian vs. Black vs. White) and feature (eyes vs. mouth vs. nose) as within-subjects independent variables. Results are shown in Fig 2.

The main effect of feature was significant, *F*(2, 156) = 243.97, *p* < .001, η_p_^2^ = .758, 95% CI [.707, .791], with participants looking at the eyes (*M* = 2363 ms, *SD* = 716 ms) longer than the nose (*M* = 887 ms, *SD* = 456 ms), *t*(80) = 12.42, *p* < .001, *d* = 1.38, 95% CI [1.07, 1.68], which was longer than the mouth (*M* = 407 ms, *SD* = 268 ms), *t*(80) = 8.49, *p* < .001, *d* = .943, 95% CI [.679, 1.20]. Most important, the interaction of feature x face race was significant, *F*(4, 312) = 2.90, *p* = .022, η_p_^2^ = .035, 95% CI [.000, .090], and did not interact with participant race, *F*(8, 312) = .734, *p* = .661, η_p_^2^ = .018, 95% CI [.000, .061], for the three-way interaction of participant race x feature x face race.

The three-way interaction was investigated further by computing two-way repeated-measures ANOVAs separately for each feature that included participant race and face race as independent variables. None of the effects were significant in the analysis of the eyes (see Fig 2, left panel), all *p*s > .052, or the nose (see Fig 2, middle panel), all *p*s > .402. The analysis of the mouth (see Fig 2, right panel) revealed a significant main effect of face race, *F*(2, 156) = 12.24, *p* < .001, η_p_^2^ = .136, 95% CI [.079, .192], with shorter longer looking times for the mouth of White faces (*M* = 358 ms, *SD* = 271 ms) than Asian faces (*M* = 424 ms, *SD* = 301 ms), *t*(80) = 3.33, *p* = .001, *d* = .370, 95% CI [.144, .595], and Black faces (*M* = 440 ms, *SD* = 275 ms), *t*(80) = 4.85, *p* < .001, *d* = .539, 95% CI [.304, .771], and no difference between Asian and Black faces, *t*(80) = 1.09, *p* = .281, *d* = .121, 95% CI [-.098, .339]. The main effect of participant race was also significant, *F*(2, 78) = 13.38, *p* < .001, η_p_^2^ = .255, 95% CI [.170, .325], with White participants looking the longest (*M* = 580 ms, *SD* = 268 ms), followed by Black participants (*M* = 379 ms, *SD* = 219 ms), *t*(51) = 2.97, *p* = .005, *d* = .816, 95% CI [.250, 1.37], which were followed by Asian participants (*M* = 260 ms, *SD* = 209 ms), *t*(51) = 2.03, *p* = .047, *d* = .559, 95% CI [.006, 1.11]. The interaction of participant race x face race was not significant, *F*(4, 156) = .420, *p* = .794, η_p_^2^ = .011, 95% CI [.000, .040].

The 3 x 3 x 3 repeated-measures ANOVA also revealed a significant interaction of participant race x feature, *F*(4, 156) = 3.40, *p* = .011, η_p_^2^ = .080, 95% CI [.032, .128], that was driven by the effect of participant race observed for the mouth but not for the eyes or nose (described above), and a significant interaction of participant race x face race, *F*(4, 156) = 2.45, *p* = .049, η_p_^2^ = .059, 95% CI [.016, .108], that was driven by overall longer looking times at Black faces than Asian or White faces by White participants. No other effects from the 3 x 3 x 3 repeated-measures ANOVA of looking times during the learning phase were significant, all *p*s > .149.

**Fig 3:**
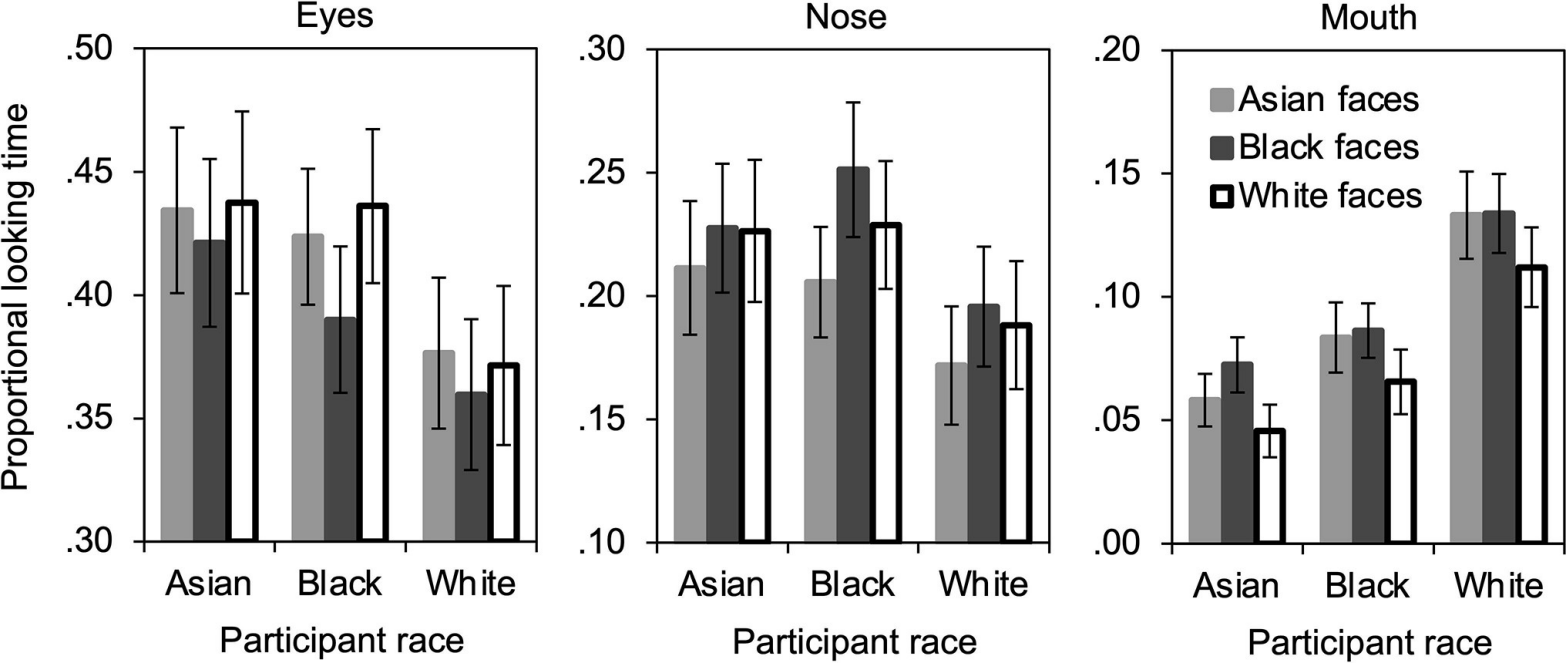
Proportional looking time during the recognition memory phase. Vertical bars indicate standard error of the mean.

Looking times during the recognition memory phase of the experiment were analyzed similarly to those from the learning phase, however, because participants’ looking times during this phase were constrained by their response times (each trial ended after the participant’s response), raw looking times (provided in Table 1) were proportionalized by response times (also provided in Table 1) prior to analysis. Proportional looking times were then analyzed in a 3 x 3 x 3 repeated-measures ANOVA in which participant race (Asian vs. Black vs. White) was a between-subjects independent variable, and face race (Asian vs. Black vs. White) and feature (eyes vs. nose vs. mouth) were within-subjects independent variables. Results are shown in Fig 3.

**Table 1:** Raw looking times and response times during the recognition memory phase.

Participant race										
Asian			Black				White			
Face race			Face race				Face race			
Asian	Black	White	Asian	Black	White	Asian		Black	White	
Eyes	874 (505)	909 (531)	892 (591)	803 (390)	781 (474)	844 (442)		817 (586)	807 (612)	787 (522)
Nose	424 (300)	492 (317)	437 (290)	367 (218)	442 (248)	428 (289)		341 (224)	394 (234)	370 (243)
Mouth	139 (130)	186 (154)	115 (131)	167 (137)	184 (159)	148 (184)		303 (250)	317 (256)	264 (219)
Response	2025 (727)	2201 (896)	2039 (985)	1892 (685)	1957 (756)	1950 (746)		2162 (1115)	2214 (1087)	2196 (1072)

‘Eyes’ indicates looking times at the eyes area. ‘Nose’ indicates looking times at the nose area. ‘Mouth’ indicates looking times at the mouth area ‘Response’ indicates response times. Parentheses indicate standard deviation of the mean. Times are provided in milliseconds.

The main effect of feature was significant, *F*(2, 156) = 92.41, *p* < .001, η_p_^2^ = .542, 95% CI [.478, .598], with longer proportional looking for the eyes (*M* = .405, *SD* = .165), than the nose (*M* = .212, *SD* = .131), *t*(80) = 6.59, *p* < .001, *d* = .732, 95% CI [.484, .975], which were longer than the mouth (*M* = .088, *SD* = .074), *t*(80) = 7.05, *p* < .001, *d* = .784, 95% CI [.533, 1.03]. The main effect of face race was also significant, *F*(2, 156) = 4.24, *p* = .016, η_p_^2^ = .052, 95% CI [.007, .099], with longer proportional looking for Black faces (*M* = .238, *SD* = .037), than Asian faces (*M* = .233, *SD* = .036), *t*(80) = 3.66, *p* < .001, *d* = .407, 95% CI [.179, .632], and White faces (*M* = .234, *SD* = .041), *t*(80) = 2.01, *p* = .048, *d* = .223, 95% CI [.002, .443], which did not differ, *t*(80) = .940, *p* = .350, *d* = .104, 95% CI [-.114, .322]. Most important, the interaction of feature x face race was significant, *F*(4, 312) = 14.54, *p* < .001, η_p_^2^ = .157, 95% CI [.104, .208], and did not interact with participant race, *F*(8, 312) = 1.35, *p* = .219, η_p_^2^ = .033, 95% CI [.000, .086], for the three-way interaction of participant race x feature x face race.

The three-way interaction was investigated further by computing two-way repeated-measures ANOVAs separately for each feature that included participant race and face race as independent variables. The analysis of the eyes (see Fig 3, left panel) revealed a main effect of face race, *F*(2, 156) = 11.61, *p* < .001, η_p_^2^ = .130, 95% CI [.074, .186], in which proportional looking times for the eyes of Black faces (*M* = .390, *SD* = .164) were shorter than those for Asian faces (*M* = .411, *SD* = .161), *t*(80) = 3.72, *p* < .001, *d* = .413, 95% CI [.185, .639], and White faces (*M* = .414, *SD* = .176), *t*(80) = 4.10, *p* < .001, *d* = .456, 95% CI [.226, .683], which did not differ, *t*(80) = .595, *p* = .553, *d* = .066, 95% CI [-.152, .284]. No other effects from the analysis of the eyes were significant, all *p*s > .116.

The analysis of the nose (see Fig 3, middle panel) revealed a main effect of face race, *F*(2, 156) = 11.73, *p* < .001, η_p_^2^ = .131, 95% CI [.075, .187], with shorter proportional looking times for the nose of Asian faces (*M* = .196, *SD* = .128) than Black faces (*M* = .224, *SD* = .135), *t*(80) = 4.60, *p* < .001, *d* = .511, 95% CI [.278, .741], and White faces (*M* = .214, *SD* = .140), *t*(80) = 3.06, *p* = .003, *d* = .340, 95% CI [.115, .563], which did not differ, *t*(80) = 1.66, *p* = .101, *d* = .184, 95% CI [-.036, .404]. No other effects from the analysis of the nose were significant, all *p*s > .345.

The analysis of the mouth (see Fig 3, right panel) revealed a main effect of face race, *F*(2, 156) = 24.47, *p* < .001, η_p_^2^ = .239, 95% CI [.179, .293], with shorter proportional looking times for the mouth of White faces (*M* = .075, *SD* = .075) than Asian faces (*M* = .092, *SD* = .181), *t*(80) = 5.93, *p* < .001, *d* = .659, 95% CI [.417, .898], and Black faces (*M* = .098, *SD* = .072), *t*(80) = 6.53, *p* < .001, *d* = .726, 95% CI [.479, .969], which did not differ, *t*(80) = 1.61, *p* = .111, *d* = .179, 95% CI [-.041, .398]. The analysis of the mouth also revealed a main effect of participant race, *F*(2, 78) = 7.06, *p* = .002, η_p_^2^ = .153, 95% CI [.069, .231], in which proportional looking times were longer for White participants (*M* = .126, *SD* = .087) than Asian participants (*M* = .058, *SD* = .055), *t*(54) = 3.51, *p* < .001, *d* = .939, 95% CI [.382, 1.49], and Black participants (*M* = .080, *SD* = .061), *t*(51) = 2.24, *p* = .029, *d* = .617, 95% CI [.062, 1.17], which did not differ, *t*(51) = 1.34, *p* = .187, *d* = .368, 95% CI [-.178, .910]. The face race x participant race interaction was not significant, *F*(4, 156) = .801, *p* = .526, η_p_^2^ = .020, 95% CI [.000, .048].

No other effects from the 3 x 3 x 3 repeated-measures ANOVA of proportional looking times during the recognition phase were significant, all *p*s > .167.

### Recognition memory

**Fig 4:**
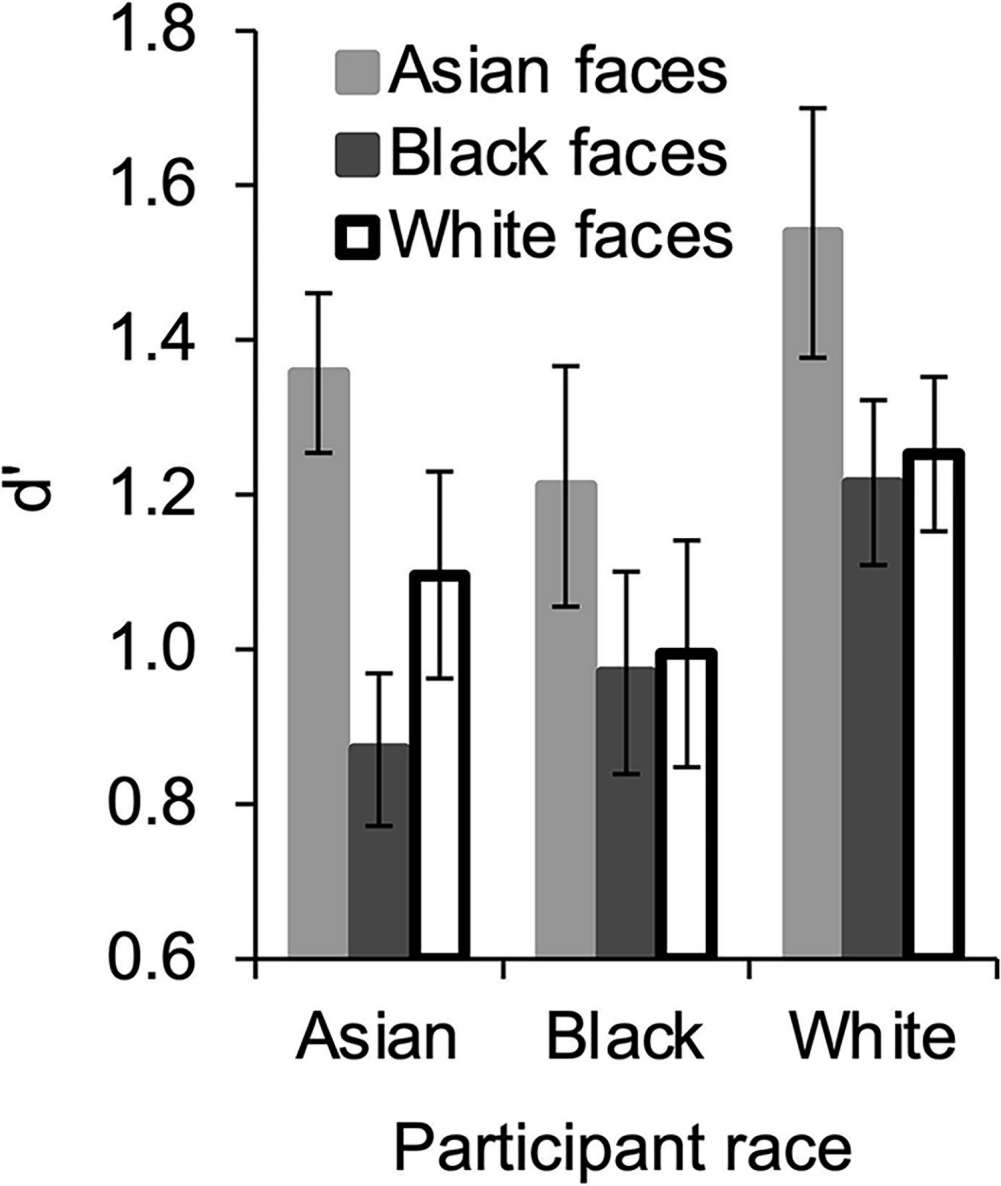
d’ During the recognition memory phase. Vertical bars indicate standard error of the mean.

Recognition memory was assessed in terms of d’, calculated as the difference between the normalized proportion of hits (correct responses that a face was presented before) minus the normalized proportion of false alarms (incorrect responses that a face was presented before). Because values of d’ are undefined when hit or false alarm rates are 1 or 0, proportions of 0 were converted to 1/(2N) and proportions of 1 were converted to 1-(1/(2N)), with N referring to the number of trials contributing to the proportion (N = 10).

d’ scores were analyzed in a 3 x 3 repeated-measures ANOVA with face race (Asian vs. Black vs. White) as a within-subjects variable and participant race (Asian vs. Black vs. White) as a between-subjects variable. Results are shown in Fig 4. The main effect of face race was significant, *F*(2, 156) = 8.92, *p* < .001, η_p_^2^ = .103, 95% CI [.052, .154], with higher d’ for Asian faces (*M* = 1.37, *SD* = .739) than Black faces (*M* = 1.02, *SD* = .591), *t*(80) = 4.24, *p* < .001, *d* = .471, 95% CI [.240, .700], and White faces (*M* = 1.12, *SD* = .660), *t*(80) = 2.94, *p* = .004, *d* = .326, 95% CI [.102, .549], which did not differ, *t*(80) = 1.17, *p* = .248, *d* = .129, 95% CI [-.090, .348]. The face race x participant race interaction was not significant, *F*(4, 156) = .450, *p* = .773, η_p_^2^ = .011, 95% CI [.000, .038], nor was the main effect of participant race, *F*(2, 78) = 2.48, *p* = .091, η_p_^2^ = .060, 95% CI [.008, .139].

In order to assess the effects of contact with different races and looking time on recognition memory, multiple-regression analyses were conducted using childhood contact and looking times to predict memory for Asian, Black, and White faces. In particular, separate analyses were performed using d’ for Asian, Black, or White faces as the dependent variable, and including as the independent variables: (1) childhood contact with Asian race; (2) childhood contact with Black race; (3) childhood contact with White race; (4) looking time at eyes for the face race being used as dependent variable (e.g., looking time at eyes of Asian faces predicting d’ for Asian faces); (5) looking time at nose for face race being used as dependent variable; and (6) looking time at mouth for face race being used as dependent variable. Childhood contact, rather than teenage contact or current contact, was used for these analyses because childhood contact has been shown to have a greater influence on the ORB than contact at older ages [21]. However, results from regression analyses using teenage contact and current contact are highly similar to those with childhood contact and are presented in S1 Table. All analyses were performed once using looking times from the learning phase and a second time using proportional looking times from the recognition memory phase.

Analyses using looking times from the learning phase revealed positive effects of nose and mouth looking on d’ for Asian faces. d’ for Asian faces increased with increased looking at the nose area, *b* = .001 (± .000), ß = .324, *t*(74) = 2.02, *p* = .047, 95% CI [.000, .001], and with increased looking at the mouth area, *b* = .001 (± .000), ß = .414, *t*(74) = 2.57, *p* = .012, 95% CI [.000, .002]. None of the other individual coefficients from these analyses reached significance, all *p*s > .081. Complete results are provided in Table 2.

**Table 2:** Results from regression analyses assessing effects of childhood contact and looking time on recognition memory.

Face race																			
			Asian						Black							White			
			*b* ±SE ß						*b* ±SE ß							*b* ±SE ß			
*Learning phase*																			
Contact Asian	.004			.007		.242		.002			.006	.176			-.002		.007	-.127	
Contact Black	.003			.008		.103		.004			.006	.159			-.003		.007	-.135	
Contact White	.003			.007		.176		.004			.006	.290			-.001		.007	-.044	
Eyes	.000			.000		.349		.000			.000	-.244			.000		.000	-.196	
Nose	.001			.000		.324*		.000			.000	-.197			.000		.000	.035	
Mouth	.001			.000		.414*		.000			.000	.176			.000		.000	-.156	
*Recognition phase*																			
Contact Asian		.005			.007	.291	.002			.006			.162	-.001			.006		-.075
Contact Black		.004			.008	.149	.004			.006			.162	-.003			.007		-.103
Contact White		.005			.007	.281	.004			.006			.288	.000			.006		-.010
Eyes		1.33			.745	.290	.443			.603			.123	-1.24			.582		-.332*
Nose		2.80			.888	.485*	.737			.685			.168	.550			.690		.117
Mouth		2.85			1.28	.312*	2.29			1.11			.280*	-1.39			1.17		-.158

* = *p* < .050.

Analyses using proportional looking times from the recognition memory phase revealed the same positive effects of nose and mouth looking on d’ for Asian faces. d’ for Asian faces increased with increased looking at the nose area, *b* = 2.80 (± .888), ß = .485, *t*(74) = 3.15, *p* = .002, 95% CI [1.03, 4.57], and increased looking at the mouth area, *b* = 2.85 (± 1.28), ß = .312, *t*(74) = 2.23, *p* = .029, 95% CI [.301, 5.39]. The analyses of proportional looking times from the recognition memory phase also revealed increased d’ for Black faces with increased looking at the mouth area, *b* = 2.29 (± 1.11), ß = .280, *t*(74) = 2.06, *p* = .043, 95% CI [.074, 4.51], and decreased d’ for White faces with increased looking at the eyes area, *b* = -1.24 (± .582), ß = -.332, *t*(74) = 2.14, *p* = .036, 95% CI [-2.40, -.084]. None of the other individual coefficients from the analyses including proportional looking times reached significance, all *p*s > .077. Complete results are provided in Table 2.

## Discussion

The present study aimed to discriminate different perspectives on the role of looking in the ORB: the perspective that the same facial features are important for memory for faces of all races and the ORB emerges when people look longer at the useful features (the eyes) for own- than other-race faces (e.g., [3–8]), versus the perspective that different facial features are useful for faces of different races and the ORB emerges when people look longer at features that are useful for their own race than at features that are useful for other-race faces (e.g., [10–14]). Overall, results are in line with the perspective that different facial features are useful for different race faces.

Most important, Asian, Black, and White participants looked at different facial features for different race faces and did so similarly regardless of their race. During the recognition memory phase, participants looked at the eyes of Asian and White faces longer than the eyes of Black faces; at the nose of Black and White faces longer than the nose of Asian faces; and at the mouth of Asian and Black faces longer than the mouth of White faces. These results are in line with previous research in which Asian and White participants looked longer at the eyes of Asian and White faces than Black faces and longer at the nose/mouth of Black faces than Asian and White faces [15], however importantly, the present study extends this finding to Black participants as well. This is critical because it clarifies whether reduced eye looking and increased nose/mouth looking for Black faces is due to them being “other race”, as was the case in Burgund [15], and as would be predicted by the perspective that the eyes are useful for all faces (e.g., [3–8]), or due to different features being useful for different race faces, as suggested by others (e.g., [10–14,16]). Clearly it is due to different features being useful for different race faces, as Black participants, for whom Black faces are “same race”, also looked less at the eyes and more at the nose and mouth of Black faces compared to Asian and White faces.

Also in line with this perspective, memory for different race faces was differentially predicted by looking at different facial features. Memory for Asian and Black faces increased with longer looking at the mouth, which complements the finding that participants looked longer at the mouth of Asian and Black faces than White faces, and strengthens support for the importance of the mouth for recognizing these race faces. Memory for Asian faces also increased with longer looking at the nose, which is somewhat surprising given that participants looked for less time at the nose of Asian faces than Black or White faces, but nonetheless underscores the differential importance of different features for Asian and Black faces. More surprisingly, memory for White faces decreased with longer looking at the eyes, which directly contradicts the perspective that the eyes are useful for recognizing all faces [3–8,15] as well as the perspective that they are useful for White faces specifically [10–14]. This negative relationship is potentially related to the lack of an ORB in the present study, which was also surprising (see below). Regardless of the reason for the effect however, when compared with the positive effects of nose and mouth looking on memory for Asian and Black faces, the negative effect of eye looking on memory for White faces further highlights the differential value of different features for different race faces.

One question about the looking results pertains to the extent to which they are affected by different sized areas of interest used for different faces and features. That is, differences in area of interest size could lead to differences in looking time simply because larger areas of interest have more opportunity to be looked at than smaller areas of interest. To control for this possibility, looking times were divided by area of interest size and reanalyzed (see S1 File). Results from these “area corrected” analyses are highly similar to those from the original “area uncorrected” analyses. Thus, differences in looking times were not due to differences in area of interest size.

Asian, Black, and White participants in the present study had different amounts of contact with Asian, Black, and White people as expected based on the countries in which they had spent their childhood and teenage years and their current college attendance in the Midwest United States. Asian participants had lived the majority of their childhood and teenage years in Asian countries in which most of the population is Asian (see Participants section), and thus, had greater contact with Asian people than Black or White people during these time periods. Almost all of the Black and White participants lived the majority of their childhood and teenage years in the United States (see Participants section), however because the population is racially segregated, Black and White participants had different amounts of contact with different races during these time periods. White participants had greater contact with White people than Asian or Black people, while Black participants had greater contact with both Black and White people than Asian people and equal contact with Black and White people. Participants’ current contact reflected their current college attendance at a predominantly White college institution located in a predominantly White neighborhood, with all participants reporting high contact with members of the White race. Critically, although childhood contact has been shown to affect the ORB in previous studies [21,22], it did not have an effect on memory in the present study. Not surprisingly, teenage contact and current contact did not affect the results either (see S1 Table).

As noted above, a surprising result from the present study was the lack of the predicted ORB. All participants, regardless of race, had better memory for Asian faces than Black or White faces and equal memory for Black and White faces. Thus, only the Asian participants exhibited an advantage for own- compared to other-race faces. This advantage seems unlikely to be due to an ORB in Asian participants however, since it was shown by Black and White participants as well. One possibility is that Asian faces in the present study were more visually distinctive from each other than Black or White faces and hence easier to discern old from new during the memory test. This possibility was examined by comparing the visual distinctiveness of faces within each race (see S2 File), however Asian faces were not more visually distinctive than Black or White faces in terms of any of the measures examined making this possibility seem less likely. Another possibility is that the greater number of female than male Asian faces (see Materials) contributed to the increased memory for Asian faces. If all the female Asian faces had higher d’ scores than male Asian faces, then the greater number of female than male faces could lead to higher d’ scores overall. An examination of the average d’ score for each face indicates that that is not the case however—female Asian faces were not uniformly higher than male Asian faces (see S2 Fig). Thus, the greater number of female than male Asian faces seems unlikely to have contributed to the higher d’ scores for Asian than Black and White faces. Other factors that have been shown to reduce or eliminate the ORB, such as motivation [27], divided attention [28], perceived similarity to oneself [29], social identity threat [30], and/or social status [31], may have influenced the present findings, however these were not measured in the present study. Of course, an optimistic view of the lack of ORB in the present study is that the effect is decreasing as racial diversity in the world increases. Unfortunately, this view is contradicted by Lee and Penrod [32] who do not observe a decrease in the ORB over time in their meta-analysis of studies published between 1969–2021. As such, we do not interpret the present lack of ORB as indicative of a larger trend.

Despite the lack of an ORB in the present study, results provide valuable insight regarding the role of looking in face processing and memory. The inclusion of Black participants in the present study is especially valuable as the vast majority of studies examining looking patterns for own and other-race faces include Asian and/or White participants [3,5–8,10,11,13–15,23,33–36], while only the Hills and Pake [12] study includes Black participants. Indeed, Black participants are rarely included in any studies of the ORB (but see [37,38]), although this is changing recently (see, e.g., [9,39–42]). As such, any eye-tracking study of the ORB that includes Black participants contributes novel information.

In sum, the present study adds to the growing amount of evidence suggesting that different facial features are useful for faces of different races. Regardless of their race, participants looked at different facial features depending on the race of the face, and different facial features were useful for memory depending on the race of the face. Although an ORB was not observed in the present study, results are in line with the perspective that the ORB emerges when people do not look at the features that are useful for faces of another race.

## Supporting information

S1 Fig Contact with different races separated by question. Vertical bars indicate standard error of the mean.
(JPG)

S2 Fig d’ Scores for individual faces. (JPG)

S1 Table Results from regression analyses with teenage and current contact. (PDF)

S1 File Results from analyses of area-corrected looking times. (PDF)

S2 File Results from analyses of visual distinctiveness. (PDF)
