# Supplementary figures and images for: Different features for different races: Tracking the eyes of Asian, Black, and White participants viewing Asian, Black, and White Faces
Source: PLoS One. 2024 Sep 18;19(9):e0310638. doi: 10.1371/journal.pone.0310638 (PMC11410263; doi:10.1371/journal.pone.0310638)

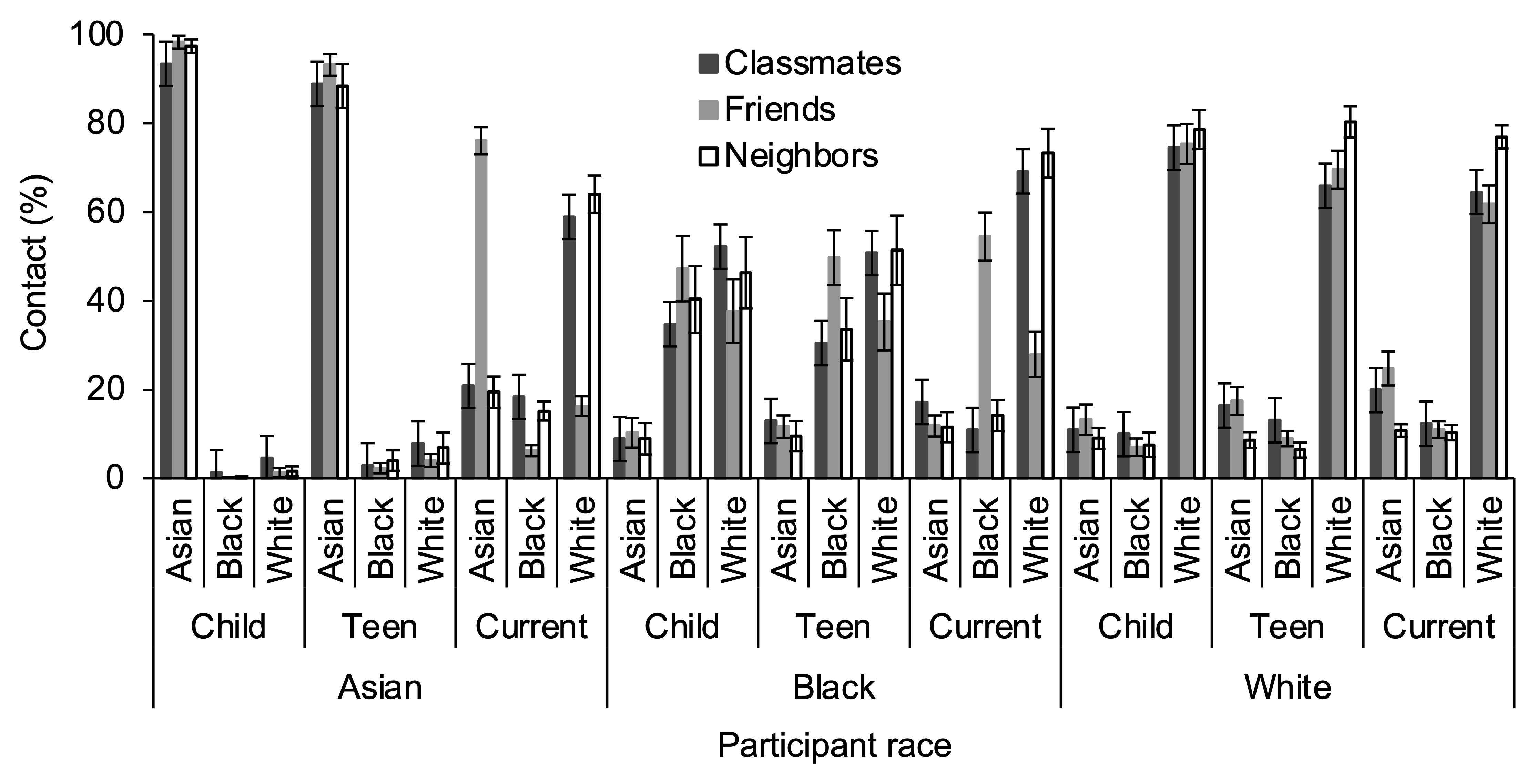

Supplement: S1 Fig — Vertical bars indicate standard error of the mean. (JPG) [file pone.0310638.s001.jpg]

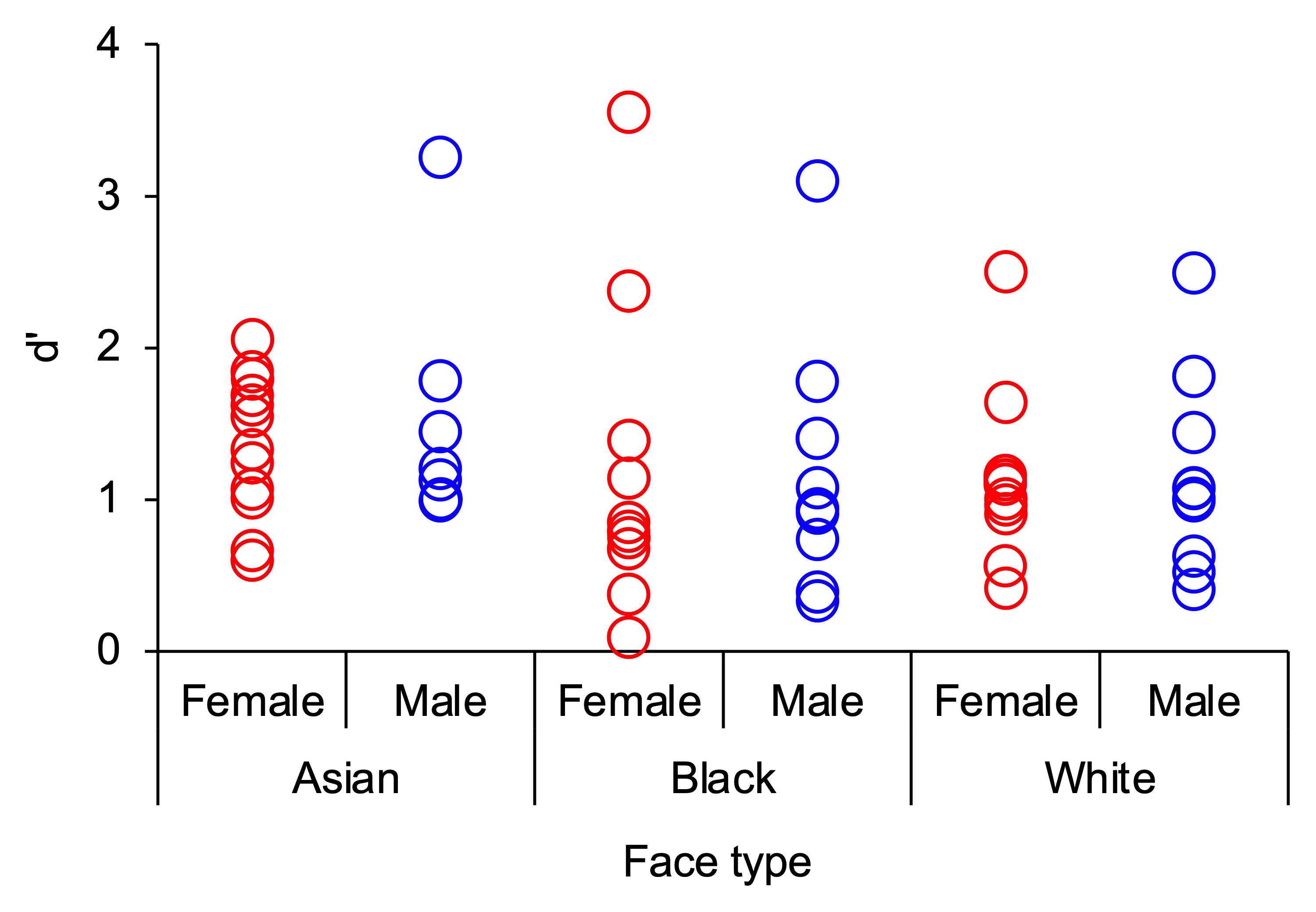

Supplement: S2 Fig — (JPG) [file pone.0310638.s002.jpg]
