# Supplementary material for: Different features for different races: Tracking the eyes of Asian, Black, and White participants viewing Asian, Black, and White Faces
Source: PLoS One. 2024 Sep 18;19(9):e0310638. doi: 10.1371/journal.pone.0310638 (PMC11410263; doi:10.1371/journal.pone.0310638)
Supplement: S1 Table — (PDF) [file pone.0310638.s003.pdf]

S1 Tables. Results from Regression Analyses with Teenage and Current Contact.

Results from Regression Analyses Assessing Effects of Teenage Contact and Looking Time on Recognition Memory.

	Face race								
	Asian			Black			White		
	b	±SE	ß	*b*	±SE	ß	*b*	±SE	ß
Learning phase									
Contact Asian	.005	.007	.255	.002	.005	.124	.002	.006	.102
Contact Black	.004	.008	.132	.003	.006	.125	.003	.007	.097
Contact White	.002	.007	.084	.003	.005	.179	.003	.007	.158
Eyes	.000	.000	.352	.000	.000	-.213	.000	.000	-.182
Nose	.001	.000	.337*	.000	.000	-.182	.000	.000	.049
Mouth	.001	.000	.464*	.000	.000	.203	.000	.000	-.171
Recognition phase									
Contact Asian	.005	.007	.286	.001	.005	.094	.001	.006	.062
Contact Black	.003	.008	.081	.003	.006	.127	.002	.007	.083
Contact White	.005	.007	.235	.004	.006	.204	.001	.006	.067
Eyes	1.40	.762	.304	.499	.616	.138	-1.28	.592	-.341*
Nose	2.85	.894	.493*	.743	.690	.169	.514	.695	.109
Mouth	3.04	1.26	.333*	2.31	1.12	.283*	-1.34	1.18	-.152

* = $p < .050$.

Results from Regression Analyses Assessing Effects of Current Contact and Looking Time on Recognition Memory.

	Face race								
	Asian			Black			White		
	b	±SE	ß	*b*	±SE	ß	*b*	±SE	ß
Learning phase									
Contact Asian	-.002	.020	-.036	-.008	.016	-.197	-.023	.018	-.488
Contact Black	-.015	.021	-.203	-.009	.017	-.155	-.033	.019	-.519
Contact White	-.014	.020	-.296	-.010	.016	-.259	-.021	.018	-.497
Eyes	.000	.000	.353	.000	.000	-.213	.000	.000	-.186
Nose	.001	.000	.334*	.000	.000	-.173	.000	.000	.017
Mouth	.001	.000	.437*	.001	.000	.251	.000	.000	-.137
Recognition phase									
Contact Asian	.001	.019	.027	-.002	.016	-.042	-.021	.017	-.463
Contact Black	-.012	.021	-.165	-.002	.017	-.027	-.032	.018	-.496
Contact White	-.012	.019	-.250	-.004	.016	-.114	-.021	.017	-.511
Eyes	1.18	.729	.257	.324	.616	.090	-1.12	.584	-.300
Nose	2.74	.860	.476*	.668	.692	.152	.585	.682	.124
Mouth	3.50	1.22	.383*	2.76	1.10	.338*	-1.34	1.11	-.152

* = $p < .050$.
