# Supplementary material for: Different features for different races: Tracking the eyes of Asian, Black, and White participants viewing Asian, Black, and White Faces
Source: PLoS One. 2024 Sep 18;19(9):e0310638. doi: 10.1371/journal.pone.0310638 (PMC11410263; doi:10.1371/journal.pone.0310638)
Supplement: S1 File — (PDF) [file pone.0310638.s004.pdf]

S1 File. Results from Analyses of Area-Corrected Looking Times.

Results from ANOVA of Area-Corrected Looking Times During the Learning Phase.

Effect	*df*	*F*
All features		
Feature	2, 156	118.57**
Feature x participant race	4, 156	3.07*
Face race	2, 156	4.05*
Face race x participant race	4, 156	2.59*
Feature x face race	4, 312	3.30*
Feature x face race x participant race	8, 312	.658
Participant race	2, 78	1.17
Eyes		
Face race	2, 156	2.31
Face race x participant race	4, 156	.917
Participant race	2, 78	3.05
Nose		
Face race	2, 156	1.01
Face race x participant race	4, 156	.990
Participant race	2, 78	.633
Mouth		
Face race	2, 156	15.58**
Face race x participant race	4, 156	.399
Participant race	2, 78	13.17**

* = $p < .050$; ** = $p < .001$.

Area-Corrected Looking Time During the Learning Phase. Values have been multiplied by 10. Vertical bars indicate standard error of the mean.

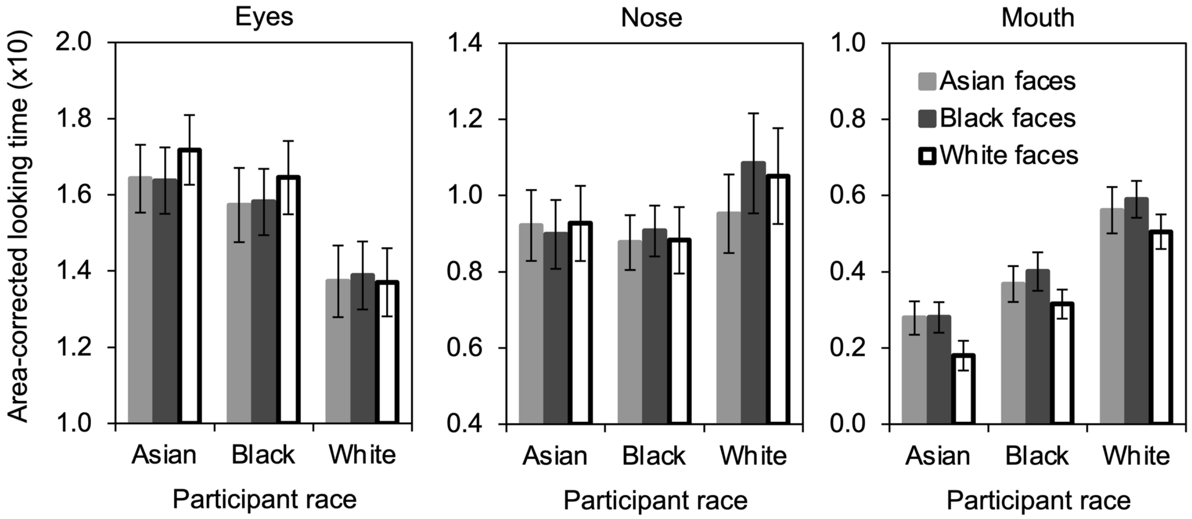

Results from ANOVA of Area-Corrected Proportional Looking Times During the Recognition Memory Phase.

Effect	*df*	*F*
All features		
Feature	2, 156	46.31**
Feature x participant race	4, 156	1.82
Face race	2, 156	22.51**
Face race x participant race	4, 156	1.26
Feature x face race	4, 312	15.90**
Feature x face race x participant race	8, 312	1.30
Participant race	2, 78	.665
Eyes		
Face race	2, 156	11.65**
Face race x participant race	4, 156	1.95
Participant race	2, 78	1.08
Nose		
Face race	2, 156	15.27**
Face race x participant race	4, 156	1.24
Participant race	2, 78	.874
Mouth		
Face race	2, 156	26.70**
Face race x participant race	4, 156	.742
Participant race	2, 78	7.03*

* = $p < .050$; ** = $p < .001$.

Area-Corrected Proportional Looking Time During the Recognition Memory Phase. Vertical bars indicate standard error of the mean.

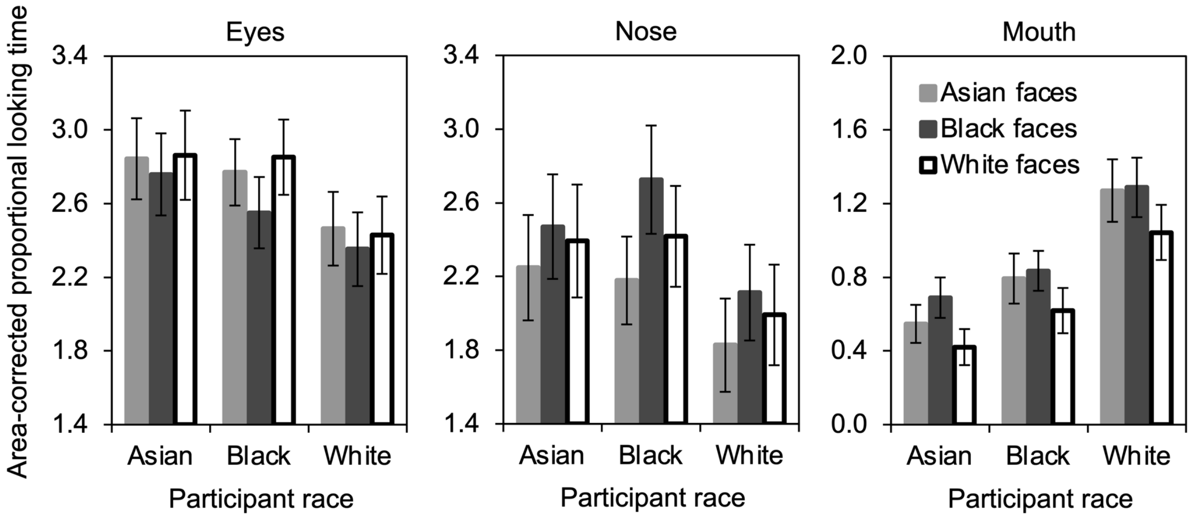

Results from Regression Analyses Assessing Effects of Childhood Contact and Area-Corrected Looking Time on Recognition Memory.

	Face race								
	Asian			Black			White		
	b	±SE	ß	*b*	±SE	ß	*b*	±SE	ß
Learning phase									
Contact Asian	.004	.007	.225	.002	.006	.216	-.002	.007	-.128
Contact Black	.003	.008	.096	.004	.006	.183	-.003	.007	-.138
Contact White	.003	.007	.164	.005	.006	.324	-.001	.007	-.054
Eyes	5.36	2.94	.357	-3.20	2.15	-.251	-2.33	2.54	-.176
Nose	5.19	2.51	.331*	-2.29	1.65	-.205	.556	2.03	.047
Mouth	10.84	4.04	.426*	3.83	3.15	.175	-3.34	4.09	-.127
Recognition phase									
Contact Asian	.005	.007	.284	.002	.006	.172	-.001	.006	-.077
Contact Black	.004	.008	.146	.004	.006	.171	-.003	.007	-.103
Contact White	.005	.007	.271	.005	.006	.296	.000	.006	-.012
Eyes	.203	.113	.288	.070	.092	.127	-.188	.089	-.329*
Nose	.265	.084	.487*	.070	.063	.172	.052	.066	.116
Mouth	.301	.133	.317*	.232	.112	.281*	-.147	.127	-.156

* = $p < .050$.

Results from Regression Analyses Assessing Effects of Teenage Contact and Area-Corrected Looking Time on Recognition Memory.

	Face race								
	Asian			Black			White		
	b	±SE	ß	*b*	±SE	ß	*b*	±SE	ß
Learning phase									
Contact Asian	.004	.007	.240	.002	.005	.154	.002	.006	.102
Contact Black	.004	.008	.130	.004	.006	.139	.003	.007	.097
Contact White	.001	.007	.068	.004	.005	.209	.003	.007	.145
Eyes	5.44	2.94	.363	-2.88	2.20	-.226	-2.16	2.57	-.163
Nose	5.43	2.53	.346*	-2.16	1.69	-.194	.737	2.07	.062
Mouth	12.23	4.21	.480*	4.30	3.42	.197	-3.68	4.39	-.141
Recognition phase									
Contact Asian	.005	.007	.282	.002	.005	.102	.001	.006	.060
Contact Black	.003	.008	.082	.004	.006	.131	.002	.007	.083
Contact White	.005	.007	.229	.004	.006	.214	.001	.006	.066
Eyes	.211	.116	.301	.079	.094	.144	-.194	.091	-.339*
Nose	.269	.084	.494*	.071	.064	.174	.048	.066	.108
Mouth	.320	.130	.337*	.234	.112	.284*	-.143	.128	-.151

* = $p < .050$.

Results from Regression Analyses Assessing Effects of Current Contact and Area-Corrected Looking Time on Recognition Memory.

	Face race								
	Asian			Black			White		
	b	±SE	ß	*b*	±SE	ß	*b*	±SE	ß
Learning phase									
Contact Asian	-.004	.019	-.075	-.008	.016	-.197	-.022	.018	-.483
Contact Black	-.016	.021	-.222	-.009	.017	-.161	-.033	.019	-.518
Contact White	-.016	.019	-.336	-.010	.016	-.258	-.021	.018	-.496
Eyes	5.40	2.85	.360	-2.93	2.13	-.230	-2.21	2.47	-.167
Nose	5.33	2.45	.339*	-2.10	1.65	-.188	.338	1.98	.028
Mouth	11.36	3.83	.446*	5.27	2.93	.241	-2.92	3.71	-.111
Recognition phase									
Contact Asian	.001	.019	.026	-.001	.016	-.023	-.022	.017	-.472
Contact Black	-.012	.021	-.163	-.001	.017	-.014	-.032	.018	-.501
Contact White	-.012	.019	-.250	-.003	.016	-.090	-.022	.017	-.522
Eyes	.178	.111	.254	.051	.094	.093	-.171	.090	-.299
Nose	.260	.081	.477*	.063	.064	.155	.054	.065	.122
Mouth	.366	.126	.385*	.278	.111	.337*	-.143	.120	-.151

* = $p < .050$.
