# Supplementary material for: Different features for different races: Tracking the eyes of Asian, Black, and White participants viewing Asian, Black, and White Faces
Source: PLoS One. 2024 Sep 18;19(9):e0310638. doi: 10.1371/journal.pone.0310638 (PMC11410263; doi:10.1371/journal.pone.0310638)
Supplement: S2 File — (PDF) [file pone.0310638.s005.pdf]

S2 File. Results from Analyses of Visual Distinctiveness.

The visual distinctiveness of faces within each race was assessed in terms of low-level visual properties and higher-order visual properties. Low-level visual properties were average luminance, extracted for each face using ImageJ (https://imagej.net/ij/), and luminance contrast, computed as the difference between the maximum and minimum luminance for each face. Higher-order visual properties were age, attractiveness, masculinity, mood, and trustworthiness taken from ratings provided by Strohminger et al. (2016). "Visual distinctiveness" values were computed for each face as the average of the absolute difference between that face and every other face in the race group divided by the value of the face, following the procedure used by Quinn et al. (2020). Values were computed in terms of each of the 7 visual properties and analyzed in separate one-way ANOVAs assessing face race (Asian vs. Black vs. White). Results are shown in the table. Critically, although the effect of face race was significant for 4 of the 7 visual properties, Asian faces were not more visually distinct than Black or White faces in any of them.

Mean Visual Distinctiveness Based on Different Properties Displayed as a Function of Face Race.

	Face race			
	Asian	Black	White	$F(2, 57)$
Luminance	.017 (.005)	.028 (.009)	.020 (.008)	13.05*
Contrast	.023 (.005)	.022 (.012)	.031 (.006)	7.87*
Age	.100 (.038)	.101 (.039)	.102 (.054)	< 1
Attractiveness	.281 (.084)	.238 (.078)	.246 (.103)	1.34
Masculinity	.417 (.070)	.636 (.109)	.554 (.065)	35.13*
Mood	.200 (.064)	.134 (.045)	.239 (.102)	10.30*
Trustworthiness	.134 (.065)	.123 (.035)	.154 (.049)	1.88

Parentheses indicate standard deviation of the mean. *F* values are from one-way ANOVAs assessing face race. * = $p < .001$.

References

Quinn, P. C., Lee, K., Pascalis, O., & Xiao, N. G. (2020). Emotional expressions reinstate recognition of other-race faces in infants following perceptual narrowing. *Developmental Psychology*, *56*(1), 15–27. https://doi.org/10.1037/dev0000858

Strohminger, N., Gray, K., Chituc, V., Heffner, J., Schein, C., & Heagins, T. B. (2016). The MR2: A multi-racial, mega-resolution database of facial stimuli. *Behavior Research Methods*, *48*(3), 1197–1204. https://doi.org/10.3758/s13428-015-0641-9
